# Cognitive functions among Saudi patients with methamphetamine and cannabis use disorder

**DOI:** 10.3389/fpsyt.2025.1621261

**Published:** 2025-08-22

**Authors:** Shrief Y. Afifi, Abdulkhaliq S. S. Alghamdi, Ahmed Sallam, Mohammed S. A. Almuqahwi, Ibrahim A. Al-sayegh, Farah M. O. Alzahrani, Amerah N. Al-Zain Al–Dain, Afrah N. Al-Zain Al-Deen, Abeer A. ALabdulhadi, Moatazbellah I. Ali

**Affiliations:** ^1^ Neuropsychiatry Department, Okasha Institute of Psychiatry, Ain Shams University, Cairo, Egypt; ^2^ Department of Addiction, Erada Complex and Mental Health in Dammam, Easter Health Cluster, Dammam, Saudi Arabia

**Keywords:** methamphetamine, cannabis, cognitive functions, MoCA, WASI, Stroop test

## Abstract

**Background:**

Methamphetamine use disorder (MUD) is linked to a variety of cognitive and neuropsychiatric deficits. One of the illegal substances that is most frequently abused is cannabis. The general consensus is that both recreational cannabis and methamphetamine use result in a wide spectrum of severe cognitive impairments, although there have been questions raised regarding conclusions derived from published material. The purpose of this work is to describe how cannabis and methamphetamine use disorder affects human cognition in a group of Saudi patients.

**Methods:**

A cross-sectional observational study has been done at Erada Complex and Mental Health, Dammam, KSA. The study included three groups: the first group consisted of individuals who have methamphetamine use disorder, the second group included individuals with disorders related to cannabis use disorder, and the third group comprised healthy persons as a control group. The patients’ demographic information has been gathered. Urine toxicological testing for cannabis and methamphetamine has been used to evaluate recent drug use. We employed SCID-I [a semi-structured interview to diagnose mental illnesses using the standards set out in the Diagnostic and Statistical Manual of Mental Disorders, Fourth Edition (DSM-IV)] to rule out other mental illnesses and confirm cannabis and methamphetamine use disorder. At the time of interview, all individuals underwent cognitive evaluations using standardized neuropsychological tests for screening by MoCA, followed by Wechsler Memory Scale and Stroop’s test for executive function.

**Results:**

A significant difference could be seen in all aspects of cognitive functions among patients and controls upon application of MoCA, Wechsler Memory Scale, and Stroop tests. On the other hand, there was statistical significance in most of the features among patients with either methamphetamine or cannabis use disorder using the same scales.

**Conclusion:**

Cognitive functions were affected in the studied group of Saudi patients who suffer from cannabis and methamphetamine use disorder compared to the control group, with those who were diagnosed with methamphetamine use disorder having greater effects on cognitive functions than those who were diagnosed with cannabis use disorder.

## 1 Introduction

Methamphetamine is a stimulant that is abused by more than 17.2 million people globally each year (1). The extent of cognitive impairments linked to methamphetamine use disorder was measured in a prior meta-analysis, which discovered that patients who are diagnosed with methamphetamine use disorder exhibit deficiencies in a number of cognitive domains, including memory consolidation, executive functioning, impulsiveness, attention, and social thinking, in comparison to healthy control subjects (2). According to Dean et al. (3), the areas with the most cognitive losses are reward- or impulse-related processes and social cognition, whereas cognitive capacity and spatial awareness show the least impact. Traditionally, methamphetamine use disorder patients have been viewed to have more neurotoxic effects than those of cannabis abuse; however, recent research has challenged this interpretation (4, 5).

According to Bernheim et al. (6), patients with methamphetamine use disorder have deficiencies in executive functioning, retention of memories, reaction suppression and set-shifting effectiveness, and psychomotor functioning. At least some of these deficits are probably related to ongoing drug use, drug pursuing, and the poor choices that come with addiction.

Several countries have updated their cannabis regulations in recent years to allow for both medicinal and recreational usage (7, 8). Concerns about how both federal and state laws affect the prevalence of cannabis usage have been raised by these shifts. More than 30 US states have authorized the use of cannabis for medical purposes, while more than 10 states have approved it for recreational consumption (9). Canada also legalized cannabis for recreational use in 2018. Evidence suggests that cannabis use disorder (CUD) and frequency of use have increased in older people (>26 years old) prior to and following medicinal and recreational regulations. There is more nuanced research on cannabis use disorder among adolescents (10, 11).

In the 1970s, when researchers first began studying the direct effects of cannabis on mental health, memory and academic issues were frequently observed. However, the findings on cognitive function have been less definitive (12, 13). According to recent studies, cannabis use disorder may be linked to long-term cognitive decline, namely, in the domains of executive functioning, memory, and visuospatial skills. These findings were also connected to changes in the cognitive functions of patients with cannabis use disorders (14). Another study illustrated that the temporal occipital fasciculus, the lateral fronto-occipital fasciculus (which plays a major role in attention and concentration), and other social cognitive regions were also shown to be affected in cannabis use disorders (15). Despite the fact that cognitive deficits in drug use disorders have been well studied in many societies (16), few studies have been conducted on Saudi patients to address this essential issue. A screening tool called the Montreal Cognitive Assessment (MoCA) assesses a number of cognitive areas, such as language, attention, memory, and visuospatial abilities. Detecting minor cognitive impairment is its intended purpose (17). Furthermore, a tool to evaluate verbal and nonverbal memory as well as perceptual–motor and cognitive capabilities is the Wechsler Memory Scale (18). Besides that, the Stroop test is a cognitive function assessment tool that evaluates selective attention, cognitive flexibility, and attentiveness. It entails identifying the ink color of words that are printed in a color that differs from the word itself (18). The purpose of the present investigation was to examine the cognitive abilities of a representative group of Saudi patients with cannabis and methamphetamine use disorder using MoCA, the Wechsler Memory Scale, and the Stroop test at Erada Complex and Mental Health in Dammam. This is crucial to develop targeted rehabilitation strategies and effective treatment plans.

## 2 Methods

### 2.1 Design and steps

This cross-sectional observational study was conducted in the Erada Complex and Mental Health in Dammam, KSA. It involved three groups: patients with methamphetamine use disorder in the first group, patients with cannabis use disorder in the second group, and healthy people in the third group as the control group. The study ran from October 2024 to March 2025.

The patients have provided demographic information such as age, sex, educational attainment, marital status, employment, and social standing. The patients’ complete medical histories have been gathered, including the duration of intake, frequency, and quantity of their cannabis and methamphetamine use as well as their past experience of other drug usage.

The inclusion criteria for the study include the following: [1] adult patients (18–50 years) who were diagnosed with methamphetamine and cannabis use disorder based on DSM-IV criteria [2]. The study included individuals with average IQ only and excluded individuals taking any medications that affect cognitive functions and individuals experiencing intoxication or withdrawal effects [3]. All of the chosen participants were fulfilling the criteria of methamphetamine and cannabis use disorder (they had at least two major criteria of substance use disorder for at least a 12-month period). On the other hand, patients with a history of other neurological or mental conditions that may impact cognitive abilities and of other drug use disorders and patients with severe illnesses that impair cognitive function, such as stroke or traumatic brain injury, were excluded from the study [4]. The sample frame that was used to include all inpatients and outpatients at the time of the research study in the addiction department at Erada Complex and Mental Health in Dammam, KSA, at the time of the study was from October 2024 to March 2025 [5]. Each patient was told of this research’s purpose and extent, and their signed informed permission was acquired. The ethical committee of the Erada Complex and Mental Health in Dammam, KSA, examined and accepted the research study (MED012). The control group included healthy individuals matched to the other two groups in sociodemographic data and their family who accepted to participate and who signed the written informed consent. MedCalc Software Ltd., Acacialaan 22, Belgium, was used to calculate the sample size. With a precision of 5% at 95% CI, the sample size was 300 participants, 100 for each group, assuming that the prevalence of amphetamine use disorder in Saudi Arabia was 10%, based on the study by Hafeiz (19), and that the average admission rate was two to three patients per day. A simple random sampling method was applied.

The exclusion criteria were as follows: [1] patients with severe illnesses that impair cognitive function, such as stroke or traumatic brain injury [2], patients with below average IQ [3], patients taking any medications known to affect cognitive functions [4], patients diagnosed to be in the intoxication or withdrawal stage of substance use, and [5] patients with substance use disorders other than methamphetamine and cannabis.

The following steps were administered among all eligible participants (based on their self-reports of drug use):

1. The Erada Complex and Mental Health psychiatry sheet includes personal information and detailed history for use disorder of cannabis and methamphetamine. A review of the coexisting medical issues was also conducted.
2. To confirm methamphetamine and cannabis use disorder and exclude other psychiatric disorders. the Structured Clinical Interview for DSM-IV Axis I Disorder (SCID-I) was used (20). A version in Arabic was employed (21). Urine toxicological assessment for cannabis and methamphetamine has been used to evaluate recent substance usage (22). At least 14 days of stoppage was needed to make sure that the participants were not in the intoxication stage and also almost nearing the end of their withdrawal symptoms.
3. Cognitive function was screened generally by the use of the Montreal Cognitive Assessment (MoCA) (23). In order to get a total score between 0 and 30, it samples behavior across 14 performance tests that include different cognitive areas. The score is expressed in integers. MoCA has shown good sensitivity and adequate specificity in detecting mild to moderate cognitive decline at a cumulative score of 25 or below (24).
4. To assess intellectual function, the Wechsler Memory Scale (WMS) assessment has been added (25). The WMS consists of four subtests: two nonverbal assessments of fluid intelligence (matrix thinking and block layout) and two verbal assessments of consolidated intelligence (vocabulary and patterns). Although they contain distinct questions, the WASI separate tests are comparable to their Wechsler Memory Scale–Third Edition equivalents (26). In order to represent a broad intellectual function or “g-factor”, the full-scale IQ (FSIQ) was chosen. Patients with marginal intellectual impairment are classified as cognitively challenged if their FSIQ is less than 86 (27).
5. The Stroop test gauges set shifting, inhibition, and attentional distortion. The Stroop examination, which has three phases, was to be administered to the participants by computer. When color circles—either blue, red, yellow, or green—appear in the center of the display screen during the first phase (color cards), the participant is instructed to press the corresponding color key on the designated Num-Lock keyboard right away. The subject must press the appropriate color key in accordance with the word without paying attention to its color in the following phase, also known as the trial phase, when a word with jumbled colors displays. Although the third stage is lengthier than the second, it is comparable to the second. The mistakes and response time are measured to grade the Stroop test.

In the incongruent condition, when the color of the ink and the word are conflicting, longer reaction times signify more cognitive interference. More mistakes in the condition that is not consistent also point to inhibition issues. The results from the Stroop test, particularly in the incongruent condition, might reveal important information about a person’s capacity to control cognitive distraction and focus (28).

### 2.2 Statistical examination

The statistical software Statistics for Social Sciences (SPSS) version 20 from IBM (SPSS Inc., Chicago, IL, USA) was used to analyze the data. Range and mean SD, as well as numbers and percentages, were used to characterize the data. By using Student’s *t*-test (T), the quantitative parameters across two groups were compared. To contrast qualitative parameters, chi-square (*χ* ^2^) and Fisher’s exact test were employed. Significance was defined as a *P*-value below 0.05. Non-significance was defined as a *P*-value above 0.05.

## 3 Results

### 3.1 Sociodemographic classification of the study group

A total of 300 participants were included in this study, with a mean age of 30.78 ± 7.78 years. The study groups included 53.33% female and 46.67% male participants. Moreover, 4.33% of the study participants have completed their primary education, 9% have been in the intermediate education level, 58.67% have completed their high school education, and 28% have a university degree. Regarding their marital status, it could be noticed that 50% of the participants were single and 36% were married. Furthermore, 61.67% of the participants do not work, and 81.33% of the tested community had a criminal record. Lastly, 66.67% of the examined group of participants were diagnosed with using either methamphetamine or cannabis, as shown in **Table 1**. The data in **Table 2** illustrate that the mean age for the control group was 33.33 ± 9.22 years, while for patients who have methamphetamine/cannabis use disorder it was 29.51 ± 6.62 years. Furthermore, the control group consisted of 78% female and 22% male participants. For patients who have methamphetamine/cannabis use disorder, it could be noticed that 41% were female and 59% were male. There is a significant difference (*P* < 0.001) among the study groups in terms of education, where the highest number of patients include those with high school education, while for the control the highest number comprised those who had a university degree. Besides that, there is a significant difference (*P* < 0.001) among the study groups in terms of marital status, where the highest number of patients included single persons, while for the control the highest number included those who were married. Additionally, the two groups had a dramatic (*P* < 0.001) difference for their work status, where the highest number for controls included those who have work, while for patients the highest number included those who do not work. Lastly, a significant difference (*P* < 0.001) could be detected among the study groups as regards their criminal record.

**Table 1:** Sociodemographic information for the whole study group.

Age		N (%)	Mean ± SD	Median (IQR)	Range
			30.78 ± 7.78	30 (25–35.5)	(18–55)
Gender	Female	160 (53.33%)			
	Male	140 (46.67%)			
Education	Illiterate	0 (0%)			
	Primary	13 (4.33%)			
	Intermediate	27 (9%)			
	High school	176 (58.67%)			
	University	84 (28%)			
Marital status	Single	150 (50%)			
	Married	108 (36%)			
	Divorced	41 (13.67%)			
	Widow	1 (0.33%)			
Job	Work	115 (38.33%)			
	Does not work	185 (61.67%)			
Criminal	No	244 (81.33%)			
	Yes	56 (18.67%)			
Group	Normal	100 (33.33%)			
	Methamphetamine/cannabis	200 (66.67%)			
Diagnosis	Normal	100 (33.33%)			
	Methamphetamine	100 (33.33%)			
	Cannabis	100 (33.33%)			

**Table 2:** Sociodemographic information between the two study groups.

		Group		Test of significance		
		Normal	Methamphetamine/cannabis			
		Mean ± SD *N* (%)	Mean ± SD *N* (%)	Value	*p*-value	Sig.
Age		33.33 ± 9.22	29.51 ± 6.62	***t*** = 3.7	<0.001	S
**Gender**	Female	78 (78%)	82 (41%)	***χ* ^2^** = 36.67	<0.001	S
	Male	22 (22%)	118 (59%)			
Education	Primary	1 (1%)^**a**^	12 (6%)^**b**^	***χ* ^2^** = 68.981	<0.001	S
	Intermediate	3 (3%)^**a**^	24 (12%)^**b**^			
	High school	38 (38%)^**a**^	138 (69%)^**b**^			
	University	58 (58%)^**a**^	26 (13%)^**b**^			
Marital status	Single	31 (31%)^**a**^	119 (59.5%)^**b**^	FE	<0.001	S
	Married	64 (64%)^**a**^	44 (22%)^**b**^			
	Divorced	4 (4%)^**a**^	37 (18.5%)^**b**^			
	Widow	1 (1%)^**a**^	0 (0%)^**a**^			
Job	Work	58 (58%)	57 (28.5%)	***χ* ^2^** = 24.543	<0.001	S
	Does not work	42 (42%)	143 (71.5%)			
Criminal	No	100 (100%)	144 (72%)	FE	<0.001	S
	Yes	0 (0%)	56 (28%)			

Each lowercase letter denotes a subset of group categories whose column proportions do not differ significantly from each other at the.05 level.

*t*, Student’s *t*-test of significance; *χ* ^2^, chi-square test of significance; FE, Fisher’s exact test of significance.

### 3.2 Evaluation of cognitive functions between study groups using different scales

Application of the MoCA test in the present work among the control group and patients who have either methamphetamine or cannabis use disorder revealed that there is a dramatic decrease (*p* < 0.001) in executive, naming, attention, language, abstraction, reminder, and total cognitive functions of the tested patients relative to the control group. Upon using the Wechsler Memory Scale assessment, two study groups reflect a significant decline (*p* < 0.001) in information, guidance, cognitive control, logic, number of repetitions, visual cues, verbal association, raw material, degree, and memory among the examined patients relative to the control group. Furthermore, using the Stroop test for measuring cognitive flexibility between two study groups revealed a significant increase (*p* < 0.001) in all examined aspects of the tested patients relative to the control group (**Table 3**).

**Table 3:** Assessment of cognitive functions among controls and patients using methamphetamine and cannabis upon using MOCA, Wechsler Memory Scale, and Stroop test for measuring cognitive flexibility (data are presented as means ± SD).

	Group		Student’s *t*-test		
	Normal	Methamphetamine/cannabis			
	Mean ± SD	Mean ± SD	*T*	*p*-value	Sig.
MOCA assessment between the two study groups					
Executive	4.45 ± 0.78	3.25 ± 1.1	10.936	<0.001	S
Naming	2.79 ± 0.41	2.34 ± 0.47	8.606	<0.001	S
Attention	5.35 ± 0.96	4.14 ± 1.35	8.970	<0.001	S
Language	2.75 ± 0.56	2.23 ± 0.85	6.325	<0.001	S
Abstraction	1.56 ± 0.5	1.1 ± 0.41	8.067	<0.001	S
Reminder	3.71 ± 1.12	1.71 ± 1.05	15.204	<0.001	S
Orientation	5.99 ± 0.1	5.19 ± 1.19	9.425	<0.001	S
Total	26.72 ± 2.03	20.34 ± 3.97	18.426	<0.001	S
Wechsler Memory Scale assessment between the two study groups					
Information	5.94 ± 0.28	5.42 ± 0.76	8.602	<0.001	S
Guidance	5 ± 0	4.19 ± 0.96	11.981	<0.001	S
Cognitive control	5.33 ± 2.01	2.74 ± 1.39	11.595	<0.001	S
Logic	16.58 ± 5.6	10.73 ± 3.98	10.430	<0.001	S
Number of repetitions	9.78 ± 1.63	7.88 ± 1.72	9.173	<0.001	S
Visual cue	11.88 ± 2.27	7.2 ± 3.05	14.942	<0.001	S
Verbal association	12.99 ± 2.28	9.07 ± 2.83	12.917	<0.001	S
Raw material	66.95 ± 9.44	47.04 ± 11.13	15.348	<0.001	S
Age	36.97 ± 3.37	35.46 ± 2.49	3.982	<0.001	S
Degree	103.86 ± 8.48	82.39 ± 10.9	18.742	<0.001	S
Memory	110.19 ± 15.07	78.89 ± 14.99	17.025	<0.001	S
Stroop test for measuring cognitive flexibility between the two study groups					
A	16.3 ± 6.42	18.22 ± 5.2	-2.782	0.006	S
B1	18.72 ± 6.94	22.1 ± 5.45	-4.255	<0.001	S
C	19.66 ± 7.69	24.18 ± 7.39	-4.926	<0.001	S
B2	35.89 ± 9.43	41.29 ± 11.93	-3.949	<0.001	S

A, for task required; B1, conflicting (yes or no); C, errors; B2, time needed.

### 3.3 Evaluation of cognitive functions between patients using drugs using different scales

Using the MoCA test in the current investigation among patients who have either methamphetamine or cannabis use disorder revealed that there is a dramatic difference (*p* < 0.001) in executive, attention, language, reminder, and total cognitive functions. Additionally, a non-significant difference could be seen in naming and abstraction (where *p* = 0.297 and 0.605, respectively) among the study groups. Upon using the Wechsler Memory Scale assessment, two study groups reflect a significant increase (*p* < 0.001) in guidance, cognitive control, logic, visual cues, verbal association, raw material, degree, and memory of patients using cannabis relative to those using methamphetamine, while there was a non-significant difference among study groups in information, number of repetitions, and age (where *p* = 0.578, 0.251, and 0.350, respectively). Furthermore, using the Stroop test to measure cognitive flexibility between the two study groups revealed a non-significant difference in all examined aspects except for B2 (for “time needed”, a significant difference could be seen among the study groups; *p* = 0.006) (**Table 4**).

**Table 4:** Assessment of cognitive functions among patients using methamphetamine or cannabis use disorders upon using MOCA, Wechsler Memory Scale, and Stroop test for measuring cognitive flexibility (data are presented as means ± SD).

	Group		Student’s *t*-test		
	Methamphetamine	Cannabis			
	Mean ± SD	Mean ± SD	*t*	*p*-value	Sig.
MOCA assessment between two drug types					
Executive	3 ± 1.06	3.49 ± 1.08	-3.236	0.001	S
Naming	2.3 ± 0.46	2.37 ± 0.49	-1.046	0.297	NS
Attention	3.7 ± 1.52	4.57 ± 1	-4.783	<0.001	S
Language	2.03 ± 0.93	2.43 ± 0.73	-3.395	0.001	S
Abstraction	1.08 ± 0.46	1.11 ± 0.35	-0.518	0.605	NS
Reminder	1.54 ± 1.17	1.88 ± 0.89	-2.316	0.022	S
Orientation	4.79 ± 1.37	5.59 ± 0.82	-5.026	<0.001	S
Total	18.8 ± 4.54	21.88 ± 2.52	-5.937	<0.001	S
Wechsler Memory Scale assessment between two drug types					
Information	5.39 ± 0.75	5.45 ± 0.77	-0.558	0.578	NS
Guidance	3.87 ± 1.04	4.5 ± 0.76	-4.890	<0.001	S
Cognitive control	2.32 ± 1.37	3.15 ± 1.29	-4.411	<0.001	S
Logic	9.99 ± 3.27	11.46 ± 4.48	-2.649	0.009	S
Number of repetitions	7.74 ± 1.79	8.02 ± 1.65	-1.152	0.251	NS
Visual cue	6.29 ± 3.02	8.11 ± 2.82	-4.410	<0.001	S
Verbal association	8.63 ± 2.64	9.51 ± 2.96	-2.219	0.028	S
Raw material	44.17 ± 9.99	49.9 ± 11.5	-3.760	<0.001	S
Age	35.62 ± 2.53	35.29 ± 2.45	0.938	0.350	NS
Degree	79.58 ± 9.72	85.19 ± 11.33	-3.758	<0.001	S
Memory	75.39 ± 13.42	82.38 ± 15.71	-3.384	0.001	S
Stroop test for measuring cognitive flexibility assessment between two drug types					
A	18.71 ± 5.69	17.73 ± 4.64	1.335	0.183	NS
B1	22.18 ± 6.38	22.02 ± 4.37	0.207	0.836	NS
C	23.41 ± 6.55	24.95 ± 8.11	-1.478	0.141	NS
B2	43.61 ± 14.45	38.97 ± 8.17	2.796	0.006	S

A, for task required; B1, conflicting (yes or no); C, errors; B2, time needed.

## 4 Discussion

The present research is designed to evaluate the cognitive abilities of Saudi patients with cannabis and methamphetamine use disorder, with a focus on certain cognitive abilities (attention, memory, and executive functioning) that are most impacted by both cannabis and methamphetamine use disorder. There is a significant decline in these functions in patients relative to the controls. Furthermore, there is a dramatic deterioration in most of the examined cognitive functions in patients with methamphetamine use disorder relative to patients with cannabis use disorder.

Methamphetamine is a psychostimulant that is frequently abused. It increases the extracellular amount of dopamine in the brain, which is linked to the rewarding effect, by reversing transit across the dopamine transporter. Substance use disorder, a continuously relapsing illness marked by obsessive drug consumption, an inability to restrict intake, and severe drug impulses, is caused by frequent methamphetamine use (29–31).

The present findings revealed that patients who have cannabis and methamphetamine use disorder had a higher risk of cognitive impairment than the controls. In accordance with other research studies, which reported that determining the degree of cognitive decline in this susceptible patient population may aid in customizing educational, career, and psychotherapy plans for them, it has been suggested that cerebral plasticity and the reconfiguration of certain brain circuits are involved in the genetic and cellular underpinnings of drug addiction, but these processes are not fully understood (32, 33). Additionally, there is growing evidence that methamphetamine and cannabis use disorder, respectively, have cognitive impairments in areas including working memory, ability to concentrate, scrutiny, social cognition, and adaptability. Additionally, patients who have cannabis and methamphetamine use disorder have changed decision-making (34, 35). The high likelihood of resurgence even after continuous abstinence with psychological assistance may be caused by cognitive disorders and impaired decision-making in methamphetamine and cannabis use disorder (36).

The present work uses different scales, including MoCA, Wechsler Memory Scale, and Stroop test, to screen the cognitive functions in the tested group of participants. Julayanont and colleagues designed the Montreal Cognitive Assessment-Basic (MoCA-B) in 2015 in order to test for moderate cognitive impairment in older individuals who are illiterate or have low levels of education (37). Furthermore, the Wechsler Memory Scale’s paired-associate learning test was used to gauge retrieval and the capacity to create new connections between two objects (38). Besides that, a variety of management, prominence, and cognitive network functions are measured using Stroop tasks (39, 40). From the lists of items written on three printed pages to be looked at as quickly as practical, the Stroop test has changed to two words on an electronic monitor with the question, “Does the color of the upper word match the concept of the lower word?”, followed by a yes/no button (41, 42).

Information, guidance, cognitive control, logic, numerical repetition, visual cues, verbal association, raw material, age, degree, and memory are some of the cognitive areas where the current findings indicate that methamphetamine and cannabis use disorder patients have worse cognition. In the same line, Potvin et al. (2) conducted a meta-analysis of users of methamphetamine use disorder and found a correlation with social cognition.

According to the current findings, consumption of methamphetamine and cognition in patients with substance use disorder was much more correlated than cannabis use disorder. In the same line, Scheffler et al. (43) showed that methamphetamine is positively correlated with cognitive functions. Besides that, according to recent meta-analyses of research on healthy populations, cannabis use disorder patients exhibit worse cognitive performance in a variety of cognitive areas when compared to non-users (44, 45). Furthermore, an investigation of longitudinal studies indicated that while consumption of cannabis was related to cognitive deterioration, the relationships were moderate, were evident primarily for the strongest users of cannabis, and were not obvious after correcting for potential confounding variables (46, 47).

Methamphetamine and cannabis use disorder patients are identified in the present investigation as risk factors for cognitive deficits in the Saudi patient group that was evaluated. Cognitive functions were affected in the studied group of Saudi patients who have cannabis and methamphetamine use disorder compared to the control group, with those who were diagnosed with methamphetamine use disorder having greater effects on cognitive functions than those who use cannabis. Future studies are needed to determine the possible neurocognitive dysfunctions in patients associated with other substance use disorders (e.g., opiates, synthetic cannabinoids, alcohol, etc.).

The study has some limitations, including the following:

1. The details of substance use were not fully assessed in depth, as they were not accurately assessed due to differences in the pattern of use regarding the amount of actual use and route of administration.
2. This study focused on a specific region in Saudi Arabia, and further studies across different regions in Saudi Arabia are needed.
3. Follow-up studies are needed to determine the long-term effect of methamphetamine and cannabis use on human cognition.

## Data Availability

The datasets presented in this study can be found in online repositories. The names of the repository/repositories and accession number(s) can be found in the article/**Supplementary Material**.
